# Monitoring Exposure to Five Chemical Warfare Agents Using the Dried Urine Spot Technique and Liquid Chromatography-Mass Spectrometry/Mass Spectrometry—In Vivo Determination of Sarin Metabolite in Mice

**DOI:** 10.3390/molecules28237687

**Published:** 2023-11-21

**Authors:** Lilach Yishai Aviram, Shai Dagan, Ariel Hindi, Shira Chapman, Rellie Gez, Eyal Drug

**Affiliations:** Department of Analytical Chemistry, Israel Institute for Biological Research (IIBR), Ness Ziona 7410001, Israel; shaid@iibr.gov.il (S.D.); arielhindi2@gmail.com (A.H.); shirac@iibr.gov.il (S.C.); relliea@iibr.gov.il (R.G.); eyald@iibr.gov.il (E.D.)

**Keywords:** sarin, soman, cyclosarin, VX, RVX, phosphonic acids, dried blood spot (DBS)

## Abstract

Dried urine spot (DUS) is a micro-sample collection technique, known for its advantages in handling, storage and shipping. It also uses only a small volume of urine, an essential consideration in working with small animals, or in acute medical situations. Alkyl-phosphonic acids are the direct and indicative metabolites of organophosphorus chemical warfare agents (OP-CWAs) and are present in blood and urine shortly after exposure. They are therefore crucially important for monitoring casualties in war and terror scenarios. We report here a new approach for the determination of the metabolites of five CWAs in urine using DUS. The method is based on a simple and rapid sample preparation, using only 50 µL of urine, spotted and dried on DBS paper, extracted using 300 µL methanol/water and analyzed via targeted LC-MS/MS. The detection limits for the five CWAs, sarin (GB), soman (GD), cyclosarin (GF), VX and RVX in human urine were from 0.5 to 5 ng/mL. Recoveries of (40–80%) were obtained in the range of 10–300 ng/mL, with a linear response (R^2^ > 0.964, R > 0.982). The method is highly stable, even with DUS samples stored up to 5 months at room temperature before analysis. It was implemented in a sarin in vivo exposure experiment on mice, applied for the time course determination of isopropyl methylphosphonic acid (IMPA, sarin hydrolysis product) in mice urine. IMPA was detectable even with samples drawn 60 h after the mice’s (IN) exposure to 1 LD_50_ sarin. This method was also evaluated in a non-targeted screening for multiple potential CWA analogs (LC-Orbitrap HRMS analysis followed by automatic peak detection and library searches). The method developed here is applicable for rapid CWA casualty monitoring.

## 1. Introduction

A chemical warfare agent (CWA) is defined as any chemical substance whose toxic properties are used to harm or incapacitate an enemy in warfare and associated military operations [1]. CWAs include five different groups of compounds: blister agents such as sulfur mustard; nerve gases including tabun, sarin and soman as the G series, and VM, RVX, VX, etc., as the V series; choking agents such as chlorine; asphyxiants (substances that cause tissue hypoxia) such as arsine; and incapacitating agents such as BZ (3-quinuclidinyl benzilate) [1]. Nerve agents are highly potent (lethal) and rapidly acting organophosphorus compounds (OPs) which produce neurotoxic effects due to their ability to act as irreversible inhibitors of the enzyme acetylcholine esterase, which is required to terminate nerve stimulation at synapses [2]. Survivors may suffer long-term effects, in particular, central and peripheral nervous system interferences affecting their neuropsychological performance. Nerve agentsexposure routes are mainly via inhalation and, for some, they are also dermal. After connecting to proteins, hydrolysis is the primary degradation pathway for many CWAs in the body. One of the nerve agents most commonly used in recent years is sarin, which was used in the Tokyo terror attack in 1995, where concentrations of 2–135 ng/mL of its degradation product isopropyl methylphosphonic acid (IMPA) were found in the blood samples of the casualties [3,4]. Sarin has also been used in the ongoing civil war in Syria [5]. VX was recently used in an assassination in Malaysia [6]. The above incidents point to the need for the detection of CWAs and their degradation products at variable concentrations, ranging from bulk to trace amounts. Monitoring body fluids may serve in forensic interrogation and the verification of individual intoxication.

GC-MS is the common analytical technique used for the detection of CWAs [7,8]. However, the hydrolysis products, as well as aqueous samples, such as body fluids, are analyzed using LC-MS(/MS), which is the first choice for the detection of biological xenobiotics, as it exhibits excellent detection limits for drugs and many other polar compounds [9]. LC-MS/MS is also the preferred tool for the quantitation of metabolites in complex matrices [10]. In particular, LC-MS/MS allows for the direct, highly sensitive and specific analysis of the polar metabolites of organophosphorus CWAs (phosphonic acids) and is superior to other analytical techniques such as GC-MS or non-MS/MS methods.

Urine is the preferred body fluid matrix for screening metabolites, drugs and other xenobiotics due to its typically higher analyte concentrations, which result in longer detection timeframes when compared to blood. In recent years, there has been increasing interest in alternative sampling methods such as dried matrix spot techniques. In these techniques, a small volume of fluid (such as blood, urine or saliva) is applied and dried on a filter paper card. This drying procedure can prevent the post-sampling formation or degradation of substances (such as those caused by bacteria) [11]. Extraction is performed directly from the filter paper and is usually very easy to accomplish. The dried urine spot (DUS) method is a promising technique since it is highly suitable for shipping and storage, and the samples are highly stable on paper [12]. Yan et al. found that the stability of their target analyte 5-MeO-DIPT was even better in DUS than in liquid urine samples [13]. This method was successfully applied in non-targeted screening for drugs of abuse [12]. Because of these advantages, as well as the fact that urine is the preferred matrix for the detection of drugs [14], the incorporation of this technique has accelerated in recent years for toxicology screening [15], metabolite-based comprehensive screening [16], forensic toxicology [17], etc. DBS was explored for the detection of exposure to CWAs, with promising results [18,19,20,21]. Although DUS has not yet been tested in this scenario, it also promises to be highly valuable in cases of large-scale field sampling, such as mass casualty events.

In this paper, we describe, for the first time, a method using DUS as a urine sample collection technique for monitoring exposure to five G- and V-type agents and demonstrate its application in a sarin in vivo experiment. The new method was optimized for targeted analysis using LC-MRM MS/MS and applied for non-target screening using LC-MS/MS (HRMS).

## 2. Results

### 2.1. Development and Optimization of the Extraction Procedure (Urine Volume, “In-Tip” Extraction Method, Solvent and Volume)

In DUS, sample volumes are usually larger than those reported for DBS for two reasons: first, accessible urine volumes are larger than blood volumes, and second, urine spreads widely over the Whatman paper [22]. To reduce dilution factors, we worked with a 50 µL urine volume, which is large enough, on the one hand, without spreading too much on the paper (around 25 mm in diameter), on the other hand.

**Figure 1:**
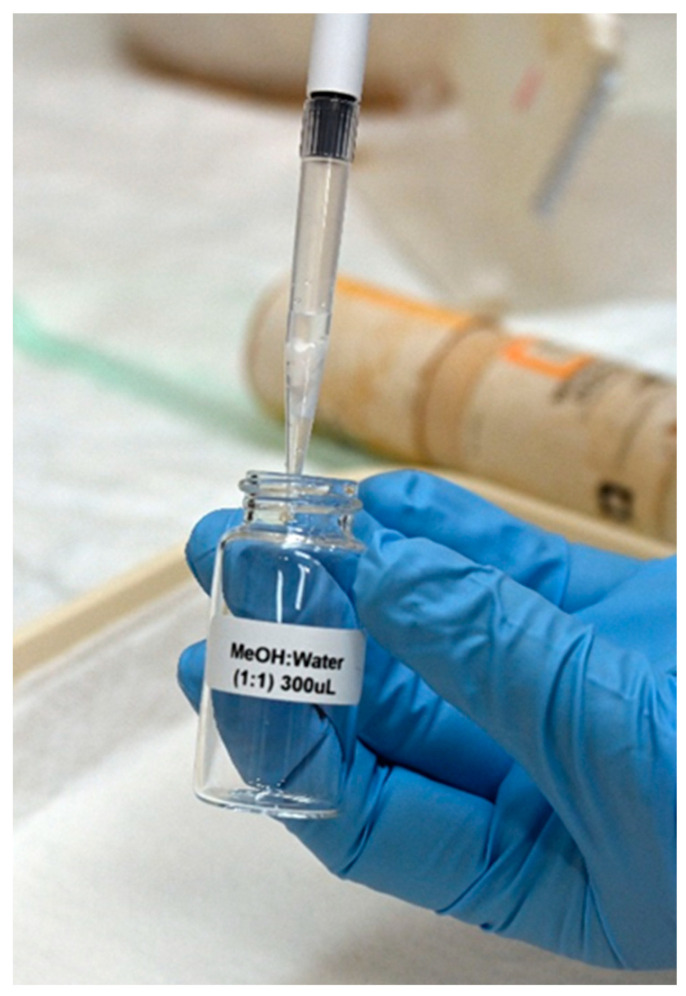
Using a polypropylene tip as a container for the extraction of the phosphonic acids from the DUS paper. The paper is “captured” on the tip. A total of 300 µL of MeOH/water was passed several times through the tip for each sample.

**Figure 2:**
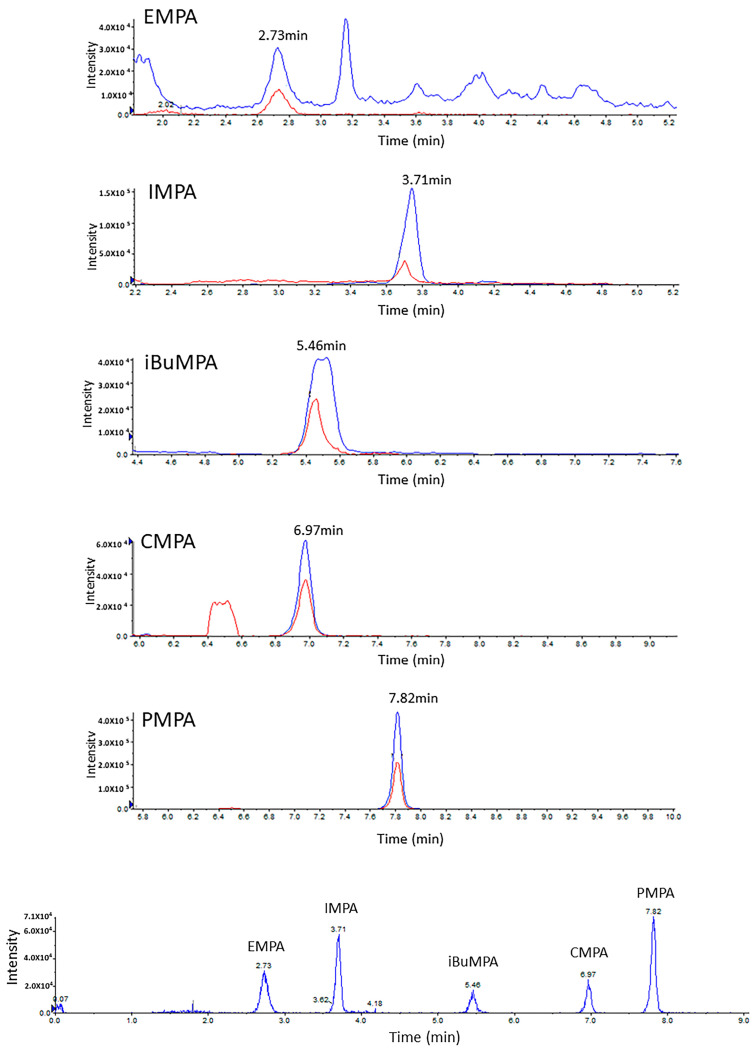
LC-MRM chromatograms of DUS samples spiked with the acids of the 5 CWAs at 5 ng/mL. Two MRM transitions (blue and red) are shown for each acid. Note that the X axes are different, separately zoomed in for each acid. In the bottom, the superposition of the 5 acids’ transitions (TIC) is shown.

In a previous study, we found that the optimized extraction solvent for phosphonic acids in DBS is a mixture of MeOH:TDW (50:50) [19]. Here, during the optimization process, several extraction volumes of this mixture (50, 100, 200, 300 and 500 µL) were evaluated. As mentioned above, since the spreading of urine on any paper is wider than that of blood due to its lower viscosity, larger extraction volumes are needed, which increase the dilution factor. To avoid this, a 1000 µL polypropylene tip was used as a “container” for the extraction processes (Figure 1). The filter paper was cut and, using disposable tweezers, placed inside the wider (upper) opening of the tip; then, the extraction solvent was uploaded into the tip (as in a normal operation, through the narrow end). The extraction solvent was then uploaded and downloaded (around 10 times) to maximize the extraction yield. Extraction using 300 µL of MeOH:H_2_O (1:1) was found to be most efficient in terms of ensuring a sufficient extract volume for analysis while maintaining a minimal dilution factor. Using the above method, two-transition MRM chromatograms with high s/n ratios were obtained at a concentration of 5 ng/mL urine (Figure 2).

### 2.2. Validation of the Method

#### 2.2.1. Calibration Model

The calibration curves of the five acids used in the DUS sample preparation procedure were obtained using the dominant transition intensity. The concentrations in the spiked urine were in the range of 0.5–300 ng/mL. The linear dynamic range obtained per acid was as follows: PMPA, 0.5–100 ng/mL (saturated at 300 ng/mL); CMPA, 1–300 ng/mL; RVX acid, 3–300 ng/mL; and IMPA and EMPA, 10–300 ng/mL. For all acids, the coefficient of determination (R^2^) was greater than 0.964 (R > 0.982). Quantitation for higher concentrations was based on the dilution of the extracts and reanalysis.

#### 2.2.2. Selectivity (Interferences)

Eight different human urine samples were used to evaluate the selectivity of the method. No interfering MRM peaks were observed at the RTs of all five phosphonic acids. However, in two human blank urine samples, there was a small peak at the MRM transition *m*/*z* 137 > 95 at 3.5 min, adjacent to the IMPA peak (3.7 min). Therefore, for IMPA, we calculated the validation parameters using the second MRM transition, 137 > 79. We examined the samples using LC-HRMS and found that the exact mass of the molecular ion of the interference at 3.5 min was different than that for the IMPA molecular ion. For all acids, compound verification was determined based on the presence of the second of the MRM transitions, with the correct intensity ratio between them.

#### 2.2.3. LOD and LOQ in Urine Samples

The LODs and LOQs are summarized in Table 1. Both were determined by spiking various concentrations to urine that was subjected to the DUS sample preparation procedure. The LODs of PMPA (soman) and CMPA (cyclosarin), which have a later t_R_ and higher molecular weights, were as low as 0.5 ng/mL, where the others were between 1 and 10 ng/mL. The higher LODs were measured for the more polar, early-eluting acids (EMPA (VX) and IMPA (sarin)), probably due to matrix effects in the earlier retention range.

#### 2.2.4. Recoveries

The recoveries were calculated at two spiked concentrations, 10 ng/mL and 100 ng/mL, for the pooled urine samples. The average recoveries ranged between 40% and 80% (Table 2). Although the recoveries were not high, they were reproducible and enabled us to obtain satisfactory LOQs, within the practical expected concentration range. 

#### 2.2.5. Accuracy and Precision

DUS calibration curves were obtained on the same day as the analytical run and served for the determination of acid concentrations. Precision and accuracy were determined at two concentration levels, 10 ng/mL and 100 ng/mL. The sample preparation procedure was repeated six times for each concentration level by two individuals. Quantitation was determined using the dominant MRM transition peak intensity for each acid, usually with a better S/N ratio. The results for accuracy and precision are summarized in Table 1.

#### 2.2.6. Stability

The stability of the acids in standard solutions (in water) is known to be good, as these materials are unreactive and thermally stable. As reported previously [23], no deterioration of the standard solutions (at room temperature, refrigeration or in the freezer) was observed. Stability tests of the acids on DUS paper were conducted in a freezer at −20 °C and in a fume hood at room temperature. For both tests, 50 µL of urine with 20 ng/mL of spiked acids was spotted onto the DUS paper. Extraction was performed every week (in triplicate each time). This experiment was carried on for five months. All peak areas and heights were compared to a fresh standard solution in water at a concentration of 20 ng/mL. We found that the DUS samples were very stable (<5% fluctuation) during these five months, both at room temperature and at −20 °C.

#### 2.2.7. Matrix Effects in LC-MS/MS

Ion suppression or enhancement is common in LC-MS with complex matrices. In this research, for all the studied acids, no major ion suppression or enhancement was evident. This was explored in five different urine samples that underwent the sample preparation procedure, spiked at the end of the process and compared to standards in water.

#### 2.2.8. DUS vs. “Dilute and Shoot”

**Figure 3:**
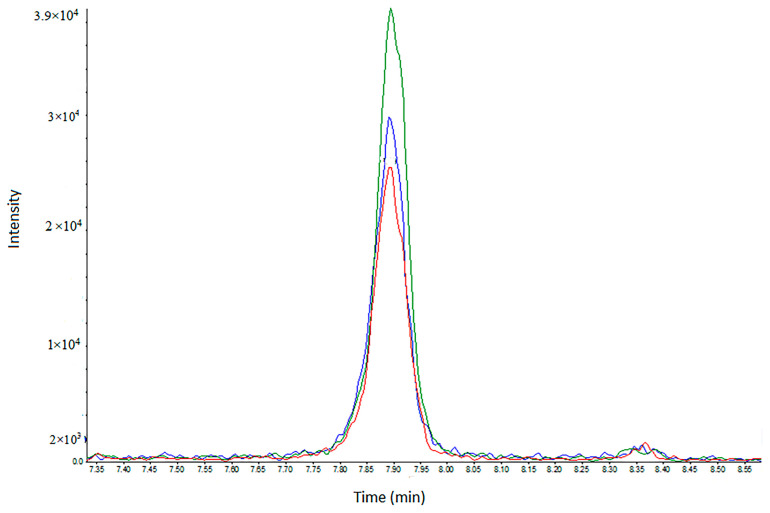
LC-MRM chromatograms of PMPA (GD acid) spiked in human urine at a concentration of 0.5 ng/mL. Red—dilute and shoot (D&S) 1:1 with water. Blue—dilute and shoot (D&S) 1:3. Green—DUS sample processing. The dominant MRM transition (179.1 > 95.0) is shown.

A successful approach in analytical toxicology is the removal of the extraction step prior to the analysis of urine, due to the desire for fast analysis. The “dilute-and-shoot’’ (D&S)–LC-MS method is the fastest strategy, being common for urine (but not for blood). Since the matrix is diluted, ion suppression is reduced [24]. This is also an excellent method for non-targeted screening, where a selective sample preparation procedure such as SPE may harm the extraction of different chemical families. Therefore, in Figure 3, we present a comparison between D&S and DUS, as demonstrated in the analysis of PMPA acid (GF). As shown before, this acid has the lowest LOD. With the DUS of a small, preserved and stable sample, the peak area is slightly higher than both the 1:1 and 1:3 D&S values of the fresh urine samples.

### 2.3. Exposure of Mice to GB—In Vivo Experiment

The new method was used to analyze IMPA obtained after the exposure of mice to sarin. For all five mice, the blank (DUS) samples, collected prior to exposure, showed no interferences at the sarin metabolite MRM transitions at the same retention time. Ion suppression was also examined by spiking 5 ng/mL of IMPA to the final DUS extract (water) and comparing it to standard solutions of IMPA in water, where the peak areas were found to be similar.

**Figure 4:**
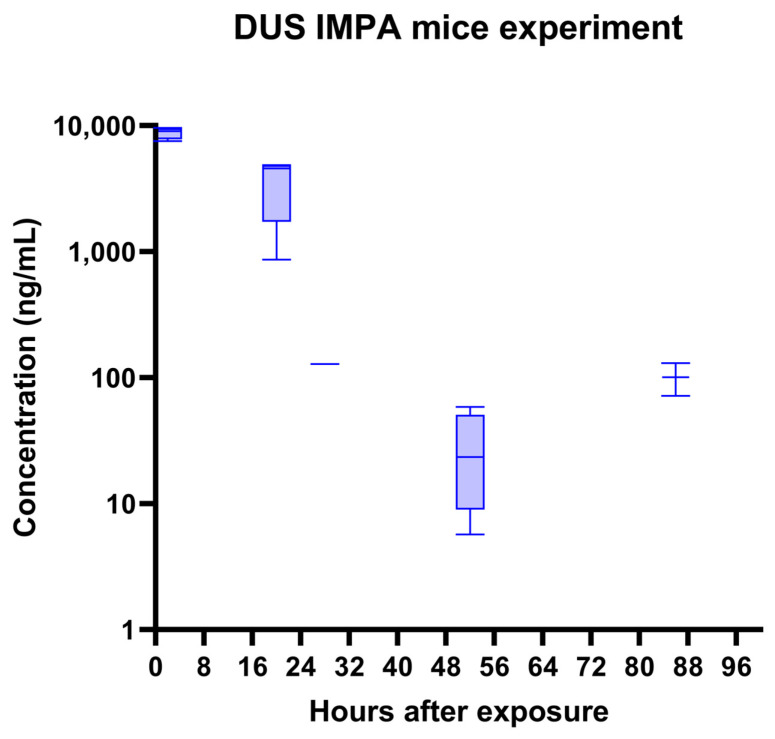
Mice’s exposure to 1LD_50_ sarin (IN). Time course of IMPA concentration in urine, as quantified using the DUS method. A single exponential decay with t_1/2_ of ~5 h is observed (except the last, 86 h point). The MRM transition (137.0 > 79.0) was used.

Figure 4 presents the kinetics of the IMPA acid concentrations in the mice urine. The concentrations were calculated according to a calibration curve of IMPA spiked in a mice urine blank. The average body weight of a mouse is around 30 g. For the 1LD50 of sarin (IV) of 150 µg/kg, the value is around 4.5 µg sarin/mouse. The estimated total volume of mouse blood is approximately 2 mL. Hence, the concentration of GB in the mouse blood immediately after exposure should be above 2 µg/mL. Hydrolysis to IMPA in the body and excretion is fast and efficient [25], which means that the concentration of IMPA in urine a few hours after exposure is expected to be in the low µg/mL range. In this study, we detected ~10 µg/mL IMPA at t = 2 h, which is a substantial portion of the initial sarin amount. We then obtained fast elimination with t_1/2_ ~5 h. These amounts and the pharmacokinetic behavior are in agreement with other findings in the literature for rabbit urine [26].

### 2.4. Non-Targeted “Screening”

**Figure 5:**
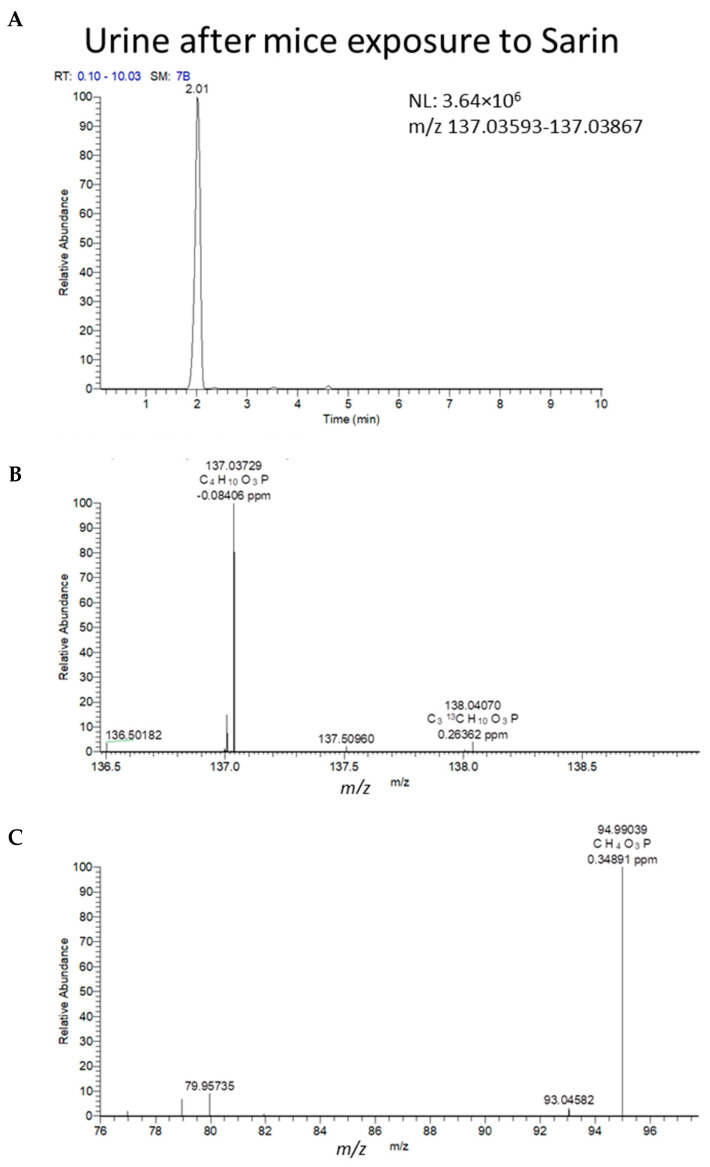
LCMS (HRMS) (negative ESI) results from DUS of mice 2 h after exposure to 1LD_50_ of sarin. (**A**) Full MS chromatogram of the molecular (MH)^−^ ion of IMPA. (**B**) MS spectra at the retention time of 2.02 min of the peak in (**A**) (*m*/*z* 137.03729 molecular ion, with the ^13^C isotopic peak at *m*/*z* 138.04070. Mass errors < 1 ppm are indicated next to each peak, relative to the theoretical *m*/*z* values. (**C**) MS/MS spectrum (NCE 35) of the same ion of *m*/*z* 137 at 2.02 min, exhibiting a product ion of IMPA at *m*/*z* 94.9904. Chemical formulae are proposed for the above ions.

While this study explored well-known targeted metabolites analyzed using LC-MRM, many other OP-CWAs may exist that have metabolites with the same phosphonate backbone but may have various modifications. A non-targeted approach should be embraced to tackle this analytical challenge. To demonstrate the potential applicability of the microsample DUS method for the detection and identification of non-targeted CWAs in urine, two in vivo mice samples were also analyzed using LC-MS (HRMS), including one that was collected 2 h after exposure and the other collected before exposure (Figure 5). The negative ESI HRMS molecular ion peak (*m*/*z* 137.037 C_4_H_10_O_3_P, with its first ^13^C isotopic peak at *m*/*z* 108.0707) and the main corresponding product ion of IMPA (*m*/*z* 94.990, CH_4_O_3_P, loss of the isopropylene group) were confirmed for the post-exposure sample. Furthermore, IMPA (serving here as an “unknown”) was detected through a full non-targeted screening protocol, using exact mass (LC-HRMS) analysis and automatic data processing with the Trace Finder software 4.1 (in its “unknown” screening mode, top 1000, negative ionization). With the in vivo DUS (2 h post-exposure), IMPA was successfully detected and identified in the NIST MS-MS library (Dot. Product 924, Rev. Dot 999). It was located high (88 out of 1000) in the intensity-sorted list of detected compounds, which makes its detection practical. We note that in this non-targeted mode, attention is naturally directed to the highest-intensity peaks (most of which represent matrix, non-toxic compounds). This makes non-targeted analysis much more challenging, with higher practical detection limits compared to targeted analysis [27]. The above finding for IMPA indicates the compatibility of the DUS method with non-targeted screening against large MS databases.

## 3. Summary and Conclusions

In a previous study [19], we reported the use of DBS for monitoring exposure to OP-CWAs. Here, for the first time, the application of DUS for the identification of CWA metabolites was explored. The above DUS and DBS methods, their performance and in vivo results (mice/rats) are compared in Table 3. The urine sample preparation is faster and simpler, and although there is no concentration step for DUS, the LODs are similar to those for blood (except for EMPA) and are appropriate for realistic scenarios. Both DBS and DUS samples may be stored at room temperature for several months (allowing convenient storage and shipping). The in vivo data, according to which much higher concentrations of IMPA were detected in urine for at least 50 h post-exposure, support the well-known preference for urine as a source for monitoring many xenobiotic materials (including direct metabolites). These DUS in vivo results demonstrate the feasibility of the method as a tool for monitoring in CWA mass casualty scenarios. Furthermore, the simplicity and robustness of the method make it easy to use, which is a major advantage in a large-scale analysis of survivors.

The DUS method was explored for targeted analysis. However, a non-targeted approach (LC-HRMS screening with automatic peak detection) was also successfully applied, providing a proof of concept for the application of DUS for the non-targeted screening of many other potential suspect OP-CWA compounds. This DUS method may be further expanded to screen for traces of other toxic materials (drugs of abuse such as synthetic opioids) and their metabolites in urine.

## 4. Materials and Methods

### 4.1. Chemicals and Reagents

The degradation products of the OP CWAs, GB, VX, GD, GF and RVX, which included isopropyl methylphosphonic acid, ethyl methylphosponic acid, pinacolyl methylphosphonic acid, cyclohexyl methylphosphonic acid, and iso-butyl methylphosphonic acid (IMPA, EMPA, PMPA, CMPA and RVX-acid), were synthesized in-house in small amounts. Methanol (MeOH) and LC-water (both ULC/MS-CC/SFC) were purchased from Bio-Lab Ltd. (Jerusalem, Israel). Ammonium formate (bioultra grade) was purchased from Sigma (St. Louis, MO, USA). The filter paper (number 1) was purchased from Whatman (Little Chalfont, UK). Pooled human urine was purchased from Elshoy Laboratories INC (ELI), (Oxford, MS, USA) and kept at 4 °C until the time of sample preparation.

### 4.2. Preparation of Standard Solutions

All stock solutions were prepared at concentrations of 2000 µg/mL in MeOH. From these solutions, the working standard solutions were prepared by diluting them with water to final concentrations ranging from 0.5 ng/mL to 2000 ng/mL. All stock solutions and standard solutions were kept at −20 °C, and before use, they were brought to room temperature. The urine was spiked with the acids (up to 3% (*v*/*v*) of the matrix solution volume). The spiked urine was used immediately after spiking.

### 4.3. Instrument Conditions

The details of the LC-MS method were reported previously and are also provided in the Supplementary Materials [19]. Briefly, a targeted analysis was performed using the LC-QQQ instrumentation A (Agilent 1290 LC coupled to a Sciex 5500 Qtrap QQQ–MS), while an untargeted analysis was performed with an LC-HRMS system (Agilent 1290 LC coupled to a Q-Exactive+MS). For the targeted analysis, the LC retention times, MRM transitions and their intensity ratios for all 5 acids are summarized in Table 4. Most of the products are common to all acids: *m*/*z* 95 is CH_4_PO_3_^−^ and *m*/*z* 79 is PO_3_^−^.

### 4.4. DUS Sample Preparation

A total of 50 µL of human urine (pre-spiked with OP-acids solution) was spotted onto the filter paper. After complete drying for approximately 2 h at room temperature, the dry urine spot was cut (25 mm in diameter, an area of approximately 4 cm^2^), folded and inserted into a polypropylene tip (1000 µL). The extraction process took place inside the tip, by uploading and downloading the extraction solvent, including 300 µL of MeOH:H_2_O, a few times. After extraction, the remaining extract volume was ~150 µL. The latter was filtered and transferred into small glass vials for LC injection without further treatment. In the in vivo experiment, mice urine was prepared similarly using the above procedure.

### 4.5. Animals

Mice (ICR) weighing 25–30 g were purchased from Envigo (Jerusalem, Israel). They were housed ten per cage in an environment with a controlled temperature of 21° ± 2 °C and a 12 h light/dark cycle with lights on at 7 a.m. Food and water were available to the mice ad lib. All procedures involving the mice were in accordance with the Guide for the Care and Use of Laboratory Animals, National Academy Press, Washington, DC, 1996, and were approved by the IIBR Animal Care and Use Committee (Protocol number M-2020).

### 4.6. In Vivo Experiment

Each mouse was located in a separate metabolic cage, and urine was collected at time intervals. In total, 12 mice were anesthetized intramuscularly with a mixture of ketamine–xylazine (1.0 and 0.1 mg/mouse, respectively), and sarin (150 µg/kg) was delivered intranasally following anesthesia. The mice were monitored for signs of toxicity 2 h post-exposure. Five mice survived, and their urine was used for the current study. Urine samples of 50 µL were collected from the mice before sarin introduction (blanks) and during the following time course: 2, 20, 28, 52 and 86 h post intoxication. The urine samples were dripped directly onto the Whatman filter paper and dried for approximately 2 h at room temperature before sample preparation and LC-MS analysis.

### 4.7. Method Validation

This method was validated for selectivity, standard curve linearity, the limit of quantitation, the limit of detection, accuracy, precision, matrix effects and stability, carryover, interferences and sample stability [28]. Following the validation criteria, the following experiment types were conducted [29]: (1) standards in water; (2) urine that underwent the sample preparation process, where the analytes were spiked in the final extract (control); and (3) spiked urine that underwent the sample preparation procedure. We used ten different batches of pooled human urine and five mice urine blanks to ensure there were no interfering/endogenous peaks at low concentrations.

The LODs were evaluated based on a criterion of signal to noise ratios ≥ 3. The LOQs were evaluated based on S/N ≥ 10. Precision and accuracy were measured at two concentrations, 10 ng/mL and 100 ng/mL, using pooled urine on three different days. The matrix effect was measured by dividing the peak area of the control sample (experiment 2) by the peak area of the standard (experiment 1). Recovery was estimated by dividing the peak area of the spiked urine sample (experiment 3) by the peak area of the control (experiment 2). Stability was examined on DUS paper at room temperature and at −20 °C for five months.

## Figures and Tables

**Table 1:** MRM transitions, t_R_, LOD and LOQ of the five phosphonic acids in urine using the DUS procedure.

Compound	Dominant MRM Transition (Negative ESI)	t_R_ (min)	LOD (ng/mL)	LOQ (ng/mL)
EMPA (VX acid)	123 > 95	2.7	10	30
IMPA (GC-acid)	137 > 79	3.7	3	9
iBuMPA (RVX-acid)	151 > 77	5.5	1	3
CMPA (GF-acid)	177 > 95	6.6	0.5	3
PMPA (GD-acid)	179 > 95	7.8	0.5	3

**Table 2:** Recovery, accuracy and precision of the new DUS method.

Compound	10 ng/mL in Urine			100 ng/mL in Urine		
	Recovery (%)	Accuracy (%)	Precision (%)	Recovery (%)	Accuracy (%)	Precision (%)
EMPA	41.7	23.1	9.2	40	21.2	12.5
IMPA	53.3	20.6	8.5	44	19.7	12.5
iBuMPA	55.5	14.6	8.9	80	12.6	11.7
CMPA	49.1	24.4	8.1	56	19.1	13.3
PMPA	54.1	16.3	8.5	54	1.9	2.9

**Table 3:** Comparison between DBS and DUS for the monitoring of CWA metabolites.

Characteristics	DBS [19]	DUS (This Study)
Sample volume, µL	20	50
Sample preparation	Extract: 400 µL MeOH Evaporation to dryness, add 400 µL water	Extract (in a polypropylene tip): 300 µL MeOH:H_2_O Filter the extract
LOD (ng/mL):		
EMPA (VX acid)	1	10
IMPA (GB—acid)	0.5	3
iBuMPA (RVX-acid)	1	1
CMPA (GF-acid)	1	0.5
PMPA (GD-acid)	0.3	0.5
Long-term sample stability on the paper	˃~1 month	˃5 months
Concentration in an in vivo experiment (ng/mL):	1LD_50,_ 5 rats, IM.:	1LD_50_, 5 mice, IN.:
2 h after the exposure (Avg)	28	8800
20 h after the exposure (Avg)		3700
24 h after the exposure (Avg)	3.3	

**Table 4:** Metabolites’ structure, formula, ionization and transitions.

Metabolite Structure	Negative Ion Formula	MRM (ESI−)	Transition Intensity Ratio
EMPA (VX Acid) 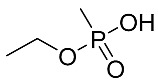	C_3_H_9_PO_3_^−^	123.0 > 95.0	3
		123.0 > 79.0	1
IMPA (GB Acid) 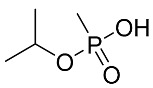	C_4_H_11_PO_3_^−^	137.0 > 95.0	6
		137.0 > 79.0	1
iBuMPA (RVX Acid) 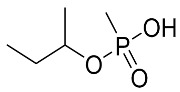	C_5_H_13_PO_3_^−^	151.1 > 77.0	2
		151.1 > 79.0	1
CMPA (GF Acid) 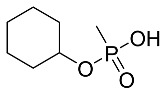	C_7_H_15_PO_3_^−^	177.1 > 95.0	2
		177.1 > 79.0	1
PMPA (GD Acid) 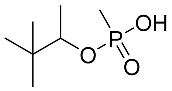	C_7_H_17_PO_3_^−^	179.1 > 95.0	2
		179.1 > 79.0	1

## Data Availability

Data are contained within the article and Supplementary Materials.

## Supplementary Materials

The following supporting information can be downloaded at: https://www.mdpi.com/article/10.3390/molecules28237687/s1, Experimental of LC-MS (QQQ) and LC-MS/MS (Orbitrap).

## References

[1] Chauhan S., D’Cruz R., Faruqi S., Singh K.K., Varma S., Singh M., Karthik V. (2008). Chemical warfare agents. Environ. Toxicol. Pharmacol..

[2] Bigley A.N., Raushel F.M. (2019). The evolution of phosphotriesterase for decontamination and detoxification of organophosphorus chemical warfare agents. Chem. Biol. Interact..

[3] Noort D., Hulst A.G., Platenburg D.H.J.M., Polhuijs M., Benschop H.P. (1998). Quantitative analysis of O-isopropyl methylphosphonic acid in serum samples of Japanese citizens allegedly exposed to sarin: Estimation of internal dosage. Arch. Toxicol..

[4] Black R.M. (2010). History and perspectives of bioanalytical methods for chemical warfare agent detection. J. Chromatogr. B..

[5] Harald J., van der Schans M.J., Koller M., Spruit E.T., Worek F., Thiermann H., Noort D. (2018). Fatal sarin poisoning in Syria 2013: Forensic verification within an international laboratory network. Forensic Toxicol..

[6] Nakagawa T., Tu A.T. (2018). Murders with VX: Aum Shinrikyo in Japan and the assassination of Kim Jong-Nam in Malaysia. Forensic Toxicol..

[7] Harald J., Thiermann H. (2021). Poisoning by organophosphorus nerve agents and pesticides: An overview of the principal strategies and current progress of mass spectrometry-based procedures for verification. J. Mass Spectrom. Adv. Clin. Lab..

[8] Marder D., Dagan S., Yishai-Aviram L., Loewenthal D., Chapman S., Adani R., Lazar S., Weissberg A., Gura S. (2020). Instantaneous monitoring of free sarin in whole blood by dry blood spot–thermal desorption–GC–FPD/MS analysis. J. Chromatogr. B.

[9] Brown H.M., McDaniel T.J., Doppalapudi K.R., Mulligan C.C., Fedick P.W. (2021). Rapid, in situ detection of chemical warfare agent simulants and hydrolysis products in bulk soils by low-cost 3D-printed cone spray ionization mass spectrometry. Analyst.

[10] Aszyk J., Byliński H., Namieśnik J., Kot-Wasik A. (2018). Main strategies, analytical trends and challenges in LC-MS and ambient mass spectrometry–based metabolomics. Trends Anal. Chem..

[11] Gaugler S., Luginbühl M., Stoeth F., Martin M., Weinmann W., König S. (2022). High resolution, high accuracy non-targeted LC-HR-MS/MS dried urine spot screening for drug of abuse testing. Toxicol. Anal..

[12] Michely J.A., Meyer M.R., Maurer H. (2018). Power of Orbitrap-based LC-high resolution-MS/MS for comprehensive drug testing in urine with or without conjugate cleavage or using dried urine spots after on-spot cleavage in comparison to established LC–MS or GC–MS procedures. Drug Test Anal..

[13] Yan X., Yuan S., Yu Z., Zhao Y., Zhang S., Wu H., Yan H., Xiang P. (2020). Development of an LC-MS/MS method for determining 5-MeO-DIPT in dried urine spots and application to forensic cases. J. Forensic Leg. Med..

[14] Pizzolato T.M., Lopez de Alda M.J., Barceló D. (2007). LC-based analysis of drugs of abuse and their metabolites in urine. Trends Anal. Chem..

[15] Pablo A., Breaud A.R., Clarke W. (2020). Automated analysis of dried urine spot (DUS) samples for toxicology screening. Clin. Biochem..

[16] Michely J.A., Meyer M.R., Maurer H. (2017). Dried urine spots—A novel sampling technique for comprehensive LC-MS^n^ drug screenin. Anal. Chim. Acta.

[17] Moretti M., Freni F., Carelli C., Previderé C., Grignani P., Vignali C., Cobo-Golpe M., Morini L. (2021). Analysis of Cannabinoids and Metabolites in Dried Urine Spots (DUS). Molecules.

[18] Yishai Aviram L., Magen M., Chapman S., Neufeld Cohen A., Lazar S., Dagan S. (2018). Dry Blood Spot sample collection for post-exposure monitoring of chemical warfare agents—In vivo determination of phosphonic acids using LC-MS/MS. J. Chromatogr. B.

[19] Yishai Aviram L., Loewenthal D., Weissberg A., Marder D., Gura S., Chapman S., Gez R., Lazar S., Dagan S. (2020). Determination of free G-type nerve agents in blood: In situ derivatization on a dried blood spot (DBS) paper followed by LC–MS/MS analysis. Forensic Toxicol..

[20] Golime R., Chandra B., Palit M., Dubey D.K. (2019). Adductomics: A promising tool for the verification of chemical warfare agents’ exposures in biological samples. Arch. Toxicol..

[21] Bruin-Hoegée M., Fidder A., van Groningen T., van der Schans M.J., Noort D., van Asten A. (2023). On-site detection and laboratory verification of the presence of nerve agent biomarkers using dried blood spots. Forensic Chem..

[22] Aramendia M., Vanhaecke F., Resano M. (2012). Direct trace-elemental analysis of urine samples by laser ablation-inductively coupled plasma mass spectrometry after sample deposition on clinical filter paper. Anal. Chem..

[23] Roen B.T., Sellevag S.R., Lundanes E. (2013). On-line solid phase extraction-liquid chromatography-mass spectrometry for trace determination of nerve agent degradation products in water samples. Anal. Chim. Acta.

[24] Deventer K., Pozo O.J., Verstraete A.G., Van Eenoo P. (2014). Dilute-and-shoot-liquid chromatography-mass spectrometry for urine analysis in doping control and analytical toxicology. Trends Anal. Chem..

[25] Roen B.T., Sellevag S.R., Lundanes E. (2014). Quantifiacation of nerve agent biomarkers in human serum and urine. Anal. Chem..

[26] Blanca M., Shifrovitch A., Dachir S., Lazar S., Elgarisi M., Prihed H., Baranes S., Egoz I., Avraham M., Dekel Jaoui H. (2021). Extended retrospective detection of regenerated sarin (GB) in rabbit blood and the IMPA metabolite in urine: A pharmacokinetics study. Arch. Toxicol.

[27] Dagan S., Marder D., Tzanani N., Drug E., Prihed H., Yishai-Aviram L. (2023). Evaluation of Matrix Complexity in Nontargeted Analysis of Small-Molecule Toxicants by Liquid Chromatography–High-Resolution Mass Spectrometry. Anal. Chem..

[28] Peters F.T., Drummer O.H., Musshoff F. (2007). Validation of new methods. Forensic Sci. Int..

[29] Matuszewski B.K., Constanzer M.L., Chavez-Eng C.M. (2003). Strategies for the assessment of matrix effect in quantitative bioanalytical methods based on HPLC–MS/MS. Anal. Chem..

