# Supplementary material for: Monitoring Exposure to Five Chemical Warfare Agents Using the Dried Urine Spot Technique and Liquid Chromatography-Mass Spectrometry/Mass Spectrometry—In Vivo Determination of Sarin Metabolite in Mice
Source: Molecules. 2023 Nov 21;28(23):7687. doi: 10.3390/molecules28237687 (PMC10707994; doi:10.3390/molecules28237687)
Supplement: Supplementary file 1 [file molecules-28-07687-s001.zip › molecules-2636862-supplementary.pdf]

Supplementary information

Experimental

LC-MS (QQQ)

Chromatographic separation was accomplished by means of an Agilent 1290 HPLC (Santa Clara, CA, USA) equipped with a C_{18} Gemini column, 3µm particle diameter, 150x2mm (Phenomenex), at 40°C. All injections were of 10µL. The mobile phase consisted of:

Gradient<br>0.3 mL/min	A: 1mM ammonium formate in LC-water (+5% methanol)	B: 1mM ammonium formate in methanol
t = 0	100%	0%
8 min	5%	95%
13 min	0%	100%
20 min	100%	0%
25 min	100%	0%

MS analysis was performed on a 5500 QTrap LIT quadrupole MS (AB Sciex, Foster city, CA, USA) in the MRM mode in negative ESI. The ion spray voltage was 4500V, ion source heater temperature 600^0 C, ion source gas 1 (air) 40 psi, ion source gas 2 (air) 60 psi, dwell time 10 msec.

LC-MS/MS (Orbitrap)

Chromatographic separation was achieved using an Agilent 1290 HPLC (Santa Clara, CA, USA), equipped with a C_{18} Gemini column, ID 3µm, 150x2mm (Phenomenex), at 40°C. All injections were of 10µL. The mobile phase consisted of 1mM of ammonium formate in LC-water (+5% methanol) at pH= 6.4 (A), and in methanol (B). The LC flow rate was 0.3 mL/min. The gradient profile was as follows: 100% A, decreased linearly to 5% between 0 and 8.00 min, then decreased further to 0% between 8.00 min to 9.5min. After 9.5min it returned to 100%, equilibrating for 4.5 min up to 14.0 min. The Orbitrap (QE+, Thermo Fisher Scientific) was equipped with a heated source operated in the positive and negative ion modes. Operating parameters were as follows: electrospray voltage, 1.25kV, sheath gas flow rate 45 (arbitrary units), auxiliary gas 10 (arbitrary units), sweep gas 2 (arbitrary units), auxiliary gas heater temperature 400 ºC, capillary temperature 275 ºC. The collision gas was nitrogen and the collision energy was set at Normalized Collision Energy (NCE) 35V.
